# Lipid Nanoparticles: A Novel Gene Delivery Technique for Clinical Application

**DOI:** 10.3390/cimb44100341

**Published:** 2022-10-19

**Authors:** Ryuichi Mashima, Shuji Takada

**Affiliations:** 1 Department of Clinical Laboratory Medicine, National Center for Child Health and Development, 2-10-1 Okura, Setagaya-ku, Tokyo 157-8535, Japan; 2 Department of Systems BioMedicine, National Research Institute for Child Health and Development, 2-10-1 Okura, Setagaya-ku, Tokyo 157-8535, Japan

**Keywords:** lipid nanoparticle, lipids, RNA modification, genome editing

## Abstract

Lipid nanoparticles (LNPs) are an emerging vehicle for gene delivery that accommodate both nucleic acid and protein. Based on the experience of therapeutic liposomes, current LNPs have been developed based on the chemistry of lipids and RNA and on the biology of human disease. LNPs have been used for the development of Onpattro, an siRNA drug for transthyretin-mediated amyloidosis, in 2018. The subsequent outbreak of COVID-19 required a vaccine for its suppression. LNP-based vaccine production received much attention for this and resulted in great success. In this review, the essential technology of LNP gene delivery has been described according to the chemistry for LNP production and biology for its clinical application.

## 1. Introduction

Lipid nanoparticles (LNPs) represent a newly emerging type of drug formulation that encapsulates biological molecules such as nucleic acids and proteins, as well as a complex made with both [1,2,3]. LNPs are spherical and are visible in electron microscopy. Therapeutic LNPs are less than 100 nm in diameter, consisting of lipids and payloads such as nucleic acids. The initial idea of LNPs stems from liposome, a much simpler lipid vesicle made with phospholipid and cholesterol and larger in size than the LNP. Liposomes were modeled based on the cell membrane under lipid bilayer theory. Liposomes have been used for research into the physical chemistry of lipids in aqueous solution and have been examined for future clinical use. For the preparation of liposomes, lipids are usually dried by a rotary evaporator, suspended within an aqueous solution, and sonicated to obtain a multi-lamellar vesicle that appears as a milky suspension.

Modern LNPs are more sophisticated and are essentially made with four different species of lipids (Table 1). The preparation procedure for LNPs may be similar to these but optimized by recent findings [4]. For LNP preparation, lipids and RNA are separately dissolved in ethanol and acidic aqueous solution, respectively. Next, they are mixed with an automated microfluidics apparatus for industrial use or a pipette for research use. Then, ethanol is removed by dialysis. In most industrial applications, several chromatographic purification procedures are required to increase the authenticity of the final LNP product. The final LNP composition is examined based on the percentage of RNA encapsulation, the diameter of the LNP, its zeta potential, and other biophysical parameters. Zeta potential represents the stability of the LNP. To optimize these acquired parameters, the polydispersity index (PDI) is used, which measures the heterogeneity of macromolecules including LNP; a value of less than 0.1 has been generally accepted as well-optimized conditions. When LNPs are formulated, lipids are used excessively over RNA (approximately 10:1 in terms of weight ratio).

LNPs in an FDA-approved formulation target the liver [2,8,9]. Upon intravenous administration, the LNPs migrate to the liver through the bloodstream, followed by incorporation into hepatocytes via an LDL receptor or an asialoglycoprotein receptor. During this migration, LNPs may associate with apolipoprotein E. Hepatic sinusoids contain numerous fenestration structures approximately 100 nm in diameter [8]. Thus, the size of the LNP needs to be smaller than this value. Note that the diameter of chylomicron, a dietary lipid droplet taken up by the intestine, is 100–1000 nm; hence, these are too large to be incorporated by the hepatocytes. In contrast, adeno-associated viruses (AAV) has an approximately 25 nm diameter; therefore, even if non-liver directed serotypes are chosen, the hepatotoxicity of AAV may become incidentally apparent.

Lastly, some previously used liposomal components are derived from naturally occurring lipids such as those originating in soy and eggs. These may contain a variety of analogs with different chain lengths and numbers of double bonds as well as isomers with different stereo- and regio-configurations. This composition may vary depending on the source of origin, diet, and even the season of harvest. To eliminate these factors, currently used lipids for LNPs are highly pure.

## 2. Lipids

**Figure 1:**
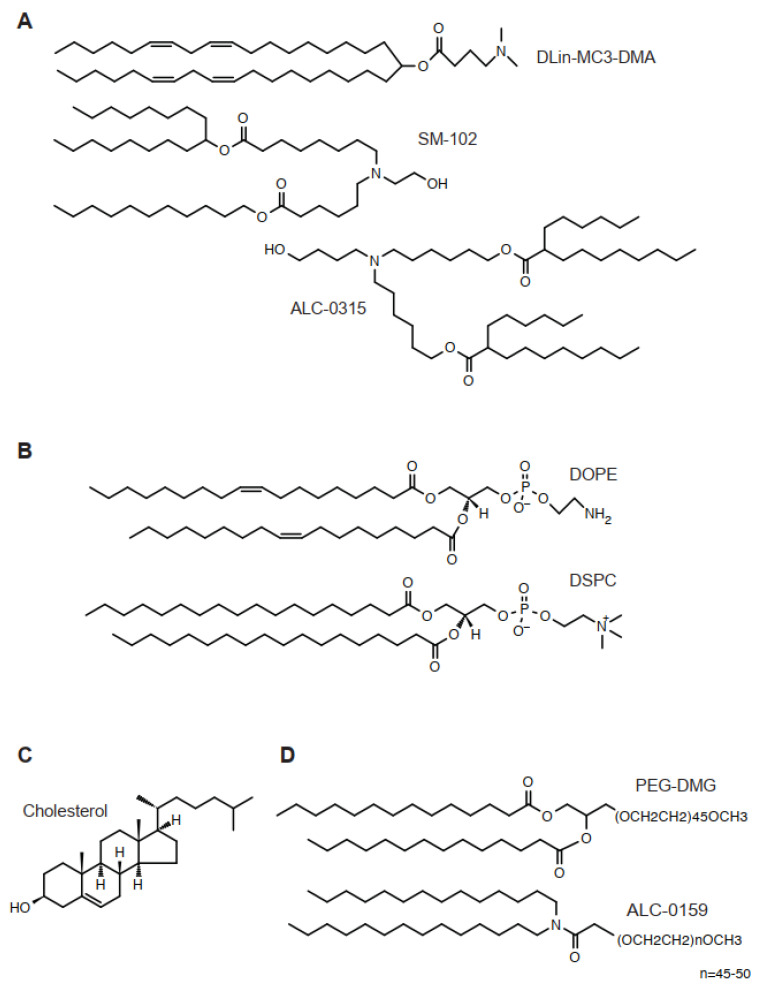
Chemical structure of lipids for LNP. (**A**) DLin-MC3-DMA, SM-102, and ALC-0315. (**B**) DOPE and DSPC. (**C**) Cholesterol. (**D**) PEG-DMG and ALC-0159.

The lipid is an essential component in LNPs, encapsulating nucleic acids and other components [10] (Figure 1). For classical formulation, four lipids are required, such as an ionizable cationic lipid, a helper lipid, cholesterol, and a PEG-lipid. The classical liposome usually required cholesterol and phospholipids, sometimes accompanied by some additional, minor components. In contrast, the molar ratio of an ionizable cationic lipid of LNPs constitutes approximately 50% of the total lipids. The best molar ratio of LNPs depends on diameter, encapsulation of nucleic acids, zeta potential, and the length of encapsulated nucleic acids. The inclusion of novel lipids requires individual optimization. The classical composition of lipids targets the liver; thus, there is a solid strategy when other tissues—such as the spleen, lung, or hematopoietic cells—are to be targeted. Below are essential components for LNPs.

### 2.1. Ionizable Cationic Lipids

An ionizable cationic lipid, such as DLin-MC3-DMA, is a tertiary amine with hydrophobic tails (Figure 1). In FDA-approved formulation, this lipid constitutes half of all lipids (Table 1). Overall, the difference of structures at the initial stage of development is often found in the linker structure, which connects the amine and hydrophobic tails. In contrast, the number of double bonds, together with their geometry, has not been discussed as a major determinant for the physical properties of LNPs [11]. Biologically degradable ionized cationic lipids tend to have many esters or thioethers.

### 2.2. Helper Lipids

DOPE and DSPC are two major phospholipids used as helper lipids in LNPs. These are used at a 10% of total lipids in mol basis. For in vivo use, DOPE seems to be preferentially used, whereas DSPC is used for in vitro use with an enhanced transfection efficiency [12,13].

### 2.3. Cholesterol

Cholesterol is an essential component of LNP. It is used at a concentration of 30–40% of total lipids depending on the molar ratio of other lipid types. Cholesterol is known to maintain LNP in a rigid form.

### 2.4. PEG-Lipids

Added PEG-lipids make up 1–5% of total lipids. At these concentrations, PEG lipids make the LNP particle much smaller. PEG lipids localize at the surface of the LNP.

## 3. RNA

RNA is an essential payload for LNP. Its efficacy is proven by the several vaccine products for COVID-19 with tremendous benefits for humans. RNA has long been thought to be an unstable biomolecule. However, an accumulating knowledge of ribonucleotides, nucleases, and the molecular mechanism of inflammation collectively is establishing an improved understanding on the stability of RNA (Table 2). Uridine is known to induce interferon production through Toll-like receptor-dependent NF-kB activation; thus, its replacement to a variety of modified uridine analogs including N1-methylpseudouridine leads to minimal cell damage in RNA-delivered cells. Among various nucleases with different substrate specificity, exonucleases have been identified as among the key factors for RNA instability. Thus, 5′-capping and/or chemical modification of minimal (usually three) nucleotides from both 5′- and 3′-end is effective for extended RNA stability. When cells are activated by RNA, an activation of RNase L, protein kinase R, and MDA5-MAVS pathways are activated, leading to apoptosis.

### 3.1. Modification of mRNA

#### 3.1.1. 5′-Capping

**Figure 2:**
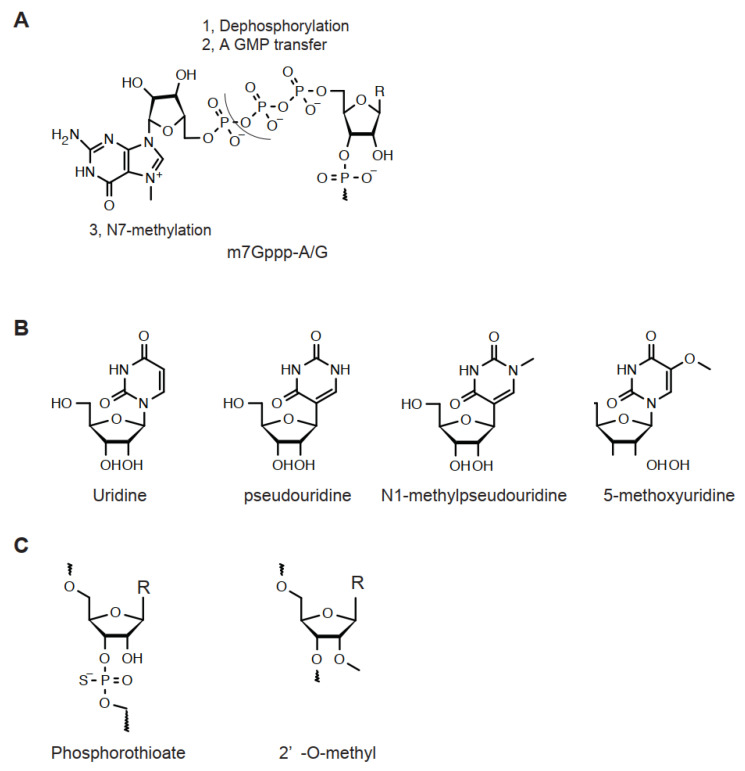
Chemical structure of ribonucleosides for LNP. (**A**) Structure of 5′-capping (m7Gppp-A/G). R = A/G. (**B**) Uridine and a series of its analog. (**C**) 2′-O-methylation and 3′/5′-O-phosphorothioation. R = A/G/U/C.

The 5′-end of mRNA is enzymatically modified and known as a 5′-capping structure (Figure 2A) [14,15]. To biochemically achieve this unique structure, a g-phosphate group of first transcription nucleoside is dephosphorylated, followed by transferring a GMP, leading to a unique 5′-Gppp-A/G structure (Figure 2A, Table 3). Then, 2,2,7-trimethylguanosine methyltransferase transfers a methyl group to the N7-position of non-first-transcribed nucleoside guanosine (Cap 0). Subsequent biochemical reactions may occur simultaneously. In Cap 1 and Cap 2 reactions, the 2′-O-position of the ribose of the first and second transcription nucleoside is methylated by 5′-capping methyltransferase 1 and 2, respectively (Cap 1 and Cap 2). In addition, an N2-methylation of guanine of non-first-transcribed nucleoside gives rise to N7, N2-dimethylated and N7, N2,2′-trimethylated guanine by 2,2,7-trimethylguanosine synthase, respectively. In parallel, N2-methylation of adenine is catalyzed by S-adenosylmethionine transferase. Once the Cap 0 structure is generated, this adenine may be further N6-methylated by an enzyme cap-specific adenosine N6-methyltransferase.

#### 3.1.2. Uridine Analog, Codon Optimization, and PolyA

In mRNA, native bases of RNA strongly activate innate immunity [16,17]. Thus, these must be modified by an analog with low immunogenicity for therapeutic purposes [18,19,20]. Uridine is a well-known example. Currently, a series of uridine analogs including pseudouridine, N1-methylpseudouridine, and 5′-methoxyuridine are used for therapeutic mRNA preparation (Figure 2B). For in vivo, N1-methylpseudouridine effectively extends the half-life of mRNA [19]. In addition, to replace uridine to its analog, an in silico procedure to reduce the number of uridines, codon optimization, may be performed. For the polyA tail, its length is correlated with mRNA stability [18].

### 3.2. Modification of Single Guide RNA (sgRNA)

The CRISPR/Cas9 system requires a guide RNA for the genome editing reaction. An sgRNA for CRISPR/Cas9 has a composed nucleotide sequence of a guide RNA and a trans-acting RNA. Usually these are used as a single strand RNA of approximately 100 nt. When extended half-life is desired, 2′-O-methylation and 5′/3′-phosphorothioation are performed [18,21,22] (Figure 2C). These modifications decrease susceptibility to exonucleases. 2′-O-fluorination is also used as an alternative modification for 2′-O-methylation.

## 4. Genetic Material

As described earlier, conventional formulation of LNPs has been demonstrated to deliver genetic material to the liver (Table 1). Such a payload includes siRNA and mRNA encoding therapeutic cDNA, Cas9 nuclease, and its related hybrid enzymes including base editor and prime editor. A ribonucleotide-protein particle (RNP) is a complex of protein and RNA with a certain stoichiometry. When the CRISPR/Cas9 system is to be used, a Cas9 nuclease forms an RNP with a guide RNA with a 1:1 molar ratio. To obtain the best results in vivo, it is desirable that the initial amount of RNA is empirically examined.

### 4.1. siRNA

Transthyretin-mediated amyloidosis is a major disease subtype of amyloidosis, and more than 30 genes have been identified as its cause [5]. While transthyretin circulates as a tetramer under normal conditions, under pathogenic conditions it is no longer metabolized properly and accumulates as amyloids in the liver. Disruption of such an accumulating pathogenic amyloid by genetic techniques is an effective treatment strategy for this disorder. Consistent with this idea, LNP-loaded siRNA for transthyretin effectively removes this [23,24]. These studies showed that, under optimized conditions, LNP-formulated siRNA for transthyretin showed high effectiveness in humans [5]. Based on this evidence, the drug Onpattro was approved by the FDA in 2018 [25]. Another example includes hypercholesterolemia, a metabolic disorder of lipids that occurs with high frequency. Pcsk9 is a serum protein that binds to an LDL receptor. Thus, an elimination of Pcsk9 followed by decreasing LDL in circulation is an effective therapy for hypercholesterolemia. As expected, such treatment effectively lowers serum LDL concentration in LNP-based models [23].

### 4.2. mRNA

#### 4.2.1. Therapeutic cDNA

Anemia is caused by an improper production of red blood cells. To remedy this, the delivery of erythropoietin mRNA to the liver has been explored [26,27,28]. The concentration of erythropoietin is significantly elevated by the LNP-mediated drug delivery system.

#### 4.2.2. Cas9 Nuclease

CRISPR/Cas9 is a leading tool for genome editing in vitro and in vivo [29,30]. Gene delivery of Cas9 in vivo needs to have some vehicle. This is readily applicable to LNP, because the size of Cas9 mRNA is approximately 4 kb. In sharp contrast, in AAV, a mainstream vector for current gene therapy, it is difficult to accommodate a wild-type Cas9 cDNA in a single vector because it is too large; hence, a two-vector system has been developed [29]. Consequently, due to the relative technical ease of Cas9 preparation, several liver disorders can be treated with LNP-mediated gene delivery. Hypercholesterolemia might be involved in one such disorder. In this case, the pathogenic Pcsk9 gene is disrupted, leading to sub-normal serum cholesterol concentration [24,31,32]. LNP-loaded Cas9 for transthyretin effectively removes this, as described above [33].

#### 4.2.3. Base Editor/Prime Editor

Adenine base editor and prime editor is an attractive research toolkit in genome editing [29]. Essentially, these are modified Cas9 with adenosine deaminase Tad7 for an adenosine base editor or reverse transcriptase for a prime editor, respectively. For rational optimization, their tertiary structure has been extensively studied [34]. As discussed above, the size of mRNA for base editors and prime editors is not suitable for a single AAV vector system. Thus, the treatment of some liver disorders has been examined using LNPs. The first application of this technology is hypercholesterolemia by targeting Pcsk9 [35,36]. Similarly, corrections of the pathogenic mutation of phenylalanine hydroxylase for phenylketonuria [37] and fumarylacetoacetate hydrolase for tyrosinemia I [38] have been examined.

## 5. Preclinical Study

### 5.1. Liver Disorders

The original formulation of LNPs preferentially target the liver. Based on this fact, liver disorders have been considered as good targets for LNP-mediated therapeutics (Table 4). Among various liver disorders, familial hypercholesterolemia (FH) is a genetic disorder involving multiple genes, such as LDLR, ApoB, and Pcsk9 [39]. LNP-based siRNA therapy has been examined for ApoB [40] and Pcsk9 [23] in murine and primate models. Pathogenic genes have also been corrected using Cas9 nuclease delivered by LNPs [24,31,32].

### 5.2. Anti-Cancer Therapy

An anti-cancer strategy based on the presentation of tumor peptides on the MHC class II by dendritic cells has been studied for more than a decade [50]. Based on the improvements of LNP delivery, its use in a conventional formulation has been explored [51]. Essentially, encapsulated mRNA for peptides of the tumor antigen was first transfected into dendritic cells, followed by administration to the melanoma model. The replacement of uridine with pseudouridine and cytidine with 5-methylcytidine reduced unselective immune activation. A more recent study reported the use of a novel amino-lipid with bio-degradative properties [52]. This LNP effectively activates CD8+ T-cells of lymph nodes in immunized mice, leading to an improving eradication of antigen-bearing melanoma in the animal model.

Near infrared (NIR) radiation-activatable LNP with a pH-sensitive dye is an emerging technique [53,54]. A recent study reported the synthesis of a novel PEG-BODIPY dye that can be used for LNP [55]. Encapsulated LNPs with this dye are incorporated into cells like other PEG-lipids, followed by the release of mRNA and the dye from the endosomal LNP into the cytosol. The released dye, rather than the PEG lipid, is then selectively activated by the NIR radiation; thus, a therapeutic outcome is expected. In this case, no targeting ligand is required for LNP, because the tumor cells are selectively killed by NIR irradiation under tumorigenic pH where this newly synthesized dye is active. These authors demonstrated that a 50:50 ratio of PEG-DMG to PEG-BODIPY dye gave the best results when examined in a subcutaneous SUM159 breast cancer xenograft model [53,56].

### 5.3. Congenital Disorders

Lysosomal storage disorders (LSDs) are characterized by an accumulation of unprocessed biochemicals in the lysosome involving 50–60 enzymes [57,58,59]. These disorders present CNS, skeletal, and visceral disorders such as hepatomegaly and splenomegaly. Fabry disease is one such LSD, with renal, cardiac, and CNS manifestations caused by pathogenic mutation of α-galactosidase A (GLA). In 2019, Zhu et al. reported that T7 RNA polymerase-transcribed therapeutic GLA mRNA with 5-methoxyuridine in LNP was administered to mice and primates, with promising results [42]. At the same time, DeRosa et al. reported that an administration of LNP-encapsulated T7 RNA polymerase-transcribed GLA mRNA with a 5′ cap structure (Cap 1) increased GLA enzyme activity more than 1000-fold compared to physiological levels [43]. More recently, Rodríguez-Castejón et al. reported that administration of a complex of plasmid DNA and protamine in LNP also provided a therapeutic effect [60]. These results raise the possibility that visceral manifestation of Fabry disease could be treated with LNP-mediated therapeutic strategies.

### 5.4. Bleeding Disorder

Factor VII is involved in the clotting process of the blood. Thus, a failure of this leads to bleeding manifestations. In the murine model, an ED50 of 0.005 mg/kg siRNA was achieved in optimized LNP composition.

### 5.5. Hemophilia A and B

Hemophilia A and B are caused by a defective activation of Factor VIII and IV, respectively, leading to improper activation of thrombin by antithrombin encoded by the *SERPINC1* gene. Thus, the degradation of this gene is expected to treat both hemophilia A and B. In a murine model, the inactivation of *Serpinc1* gene by LNP-encapsulated siRNA has been examined with a therapeutic outcome.

### 5.6. Duchenne Muscular Dystrophy (DMD)

DMD is a muscular disorder. DMD in approximately 10% of affected individuals is caused by pathogenic exon skipping. In these cases, an alteration of this followed by restoration of wild-type reading frame is known to be therapeutic in DMD even if some amino acids are still missing. Based on this fact, an originally established murine DMD model harboring human DMD exon 45 by a knock-in strategy was corrected by LNP-encapsulated Cas9 mRNA.

### 5.7. Human Immunodeficiency Virus (HIV)

HIV is a member of the Retroviridae family, which causes human immunodeficiency. Antibody-mediated neutralization of HIV is one effective therapeutic strategy. N. Pardi et al. administered LNP-encapsulated mRNA for both light and heavy chains of monoclonal antibody against HIV. An administration of 1.4 mg/kg of mRNA into mice resulted in an approximately 170 mg/mL monoclonal antibody. The efficacy of this treatment was further demonstrated with the intravenous HIV-1-challenge in a humanized HIV murine model.

## 6. Clinical Study

According to the results of preclinical studies, more than 10 clinical studies are now ongoing (Table 5). Most notably, the clinical study of LNP-encapsulated siRNA for transthyretin has been completed, and this agent was approved by the FDA in 2018. Similar to this success, a clinical trial of genome-editing therapy is ongoing. Apart from these, other trials mainly involve anti-cancer therapy. The administration of an LNP-encapsulated payload is delivered into the body either intravenously or intratumorally. Among many cancers, most are liver cancers followed by lung cancers, consistent with the effectiveness of LNP for liver-targeted delivery vehicle.

### 6.1. Transthyretin

#### 6.1.1. siRNA

Patisiran is an approved RNA-interference therapeutic for hereditary transthyretin amyloidosis, a type of amyloidosis with a high prevalence of known etiology among more than 30 genes. The study was performed with patients with hereditary transthyretin amyloidosis at 0.3 mg/kg once every 3 weeks; [5,62]. Patients aged between 18 and 85 years old with a diagnosis of hATTR were recruited from 44 sites across 19 countries [5,62] and received Patisiran at 0.3 mg/kg intravenously once every 3 weeks for 18 months. Under this condition, the mean changes in serum transthyretin concentration decreased at 3 weeks. At 18 months, Patisiran improved multiple manifestations such as motor, sensory, and autonomic neuropathy collectively measured with an index called the Neuropathy Impairment Score+7 (mNIS + 7). Overall, other parameters including cardiac function and quality of life were improved compared to controls [56,63,64].

#### 6.1.2. Cas9 Nuclease

The results of Phase I of the clinical trial of CRISPR-Cas9-mediated in vivo gene editing for transthyretin amyloidosis (NTLA-2001) was recently reported [61]. In this study, an observation of the first 28 days of treatment at 0.1–0.3mg/kg reported few adverse events with a reduction of mean serum transthyretin concentration at 52% at 0.1 mg/kg (*n* = 3) and 87% at 0.3 mg/kg (*n* = 3) in humans (NCT04601051). This clinical study was designed based on the preclinical study in the non-human primates at 1.5–6.0 mg/kg with no adverse events up to 12 months. There were seven putative targeting sites for this therapeutic CRISPR-Cas9 system. The authors reported that all these loci were located in non-coding regions as determined using established software such as Cas-OFFinder, GUIDE-seq, and SITE-seq, and there was no evidence of off-targeting when examined using primary human hepatocytes with a three-fold higher concentration of NTLA-2001.

### 6.2. Solid Tumor

Checkpoint inhibition through an interaction between PD-1/PD-L1 and CTLA-4 plays a key role in activating the tumor-killing activity of T cells [66]. Therefore, therapeutic cancer vaccines educate the immune system of tumor-bearing patients in response to individual tumor-specific immunity. A clinical study based on this mechanism using LNP as a vehicle for therapeutic mRNA is now ongoing (NCT03739931) [66].

### 6.3. Hepatic Fibrosis

Hepatic fibrosis is caused by a failure to maintain the balance between a wound process caused by inflammation and the subsequent healing process in the liver. A Phase 1b/2 clinical trial has been performed. In this study, 25 participants with moderate to extensive hepatic fibrosis were enrolled, and the safety and tolerability of LNP-encapsulated siRNA for HSP47 was examined (NCT02227459). The siRNA agent was administered to the subjects aged between 18 to 75 years old once or twice a week for 5 weeks.

### 6.4. Ornithine Transcarbamylase Deficiency (OTCD)

OTCD is an inherited metabolic disorder in the liver. The deficiency of ornithine transcarbamylase fails to synthesize citrulline at the second step of urea cycle, leading to hyperammonemia. Administration of therapeutic mRNA for ornithine transcarbamylase has been examined (NCT04416126/NCT04442347/NCT05526066). In a Phase 1 trial, 30 subjects aged between 18 to 65 years old were enrolled for a single ascending dose study with no reported adverse events.

## 7. Tissue Targeting of LNP

Tissue targeting is another issue for LNPs. It is well known that the initial formulation of LNP effectively delivers the payload to the liver (Table 1). Consistently, accumulating examples in preclinical and clinical trial support this evidence (Table 4 and Table 5). For future expansion of LNP as the vehicle of a drug delivery system, numerous studies are been currently performed. Actually, such a systematic project has been known. Selective organ targeting (SORT) is an idea for tissue targeting of LNPs proposed by Siegwart et al. [11,31,67,68,69] (Table 6). These authors hypothesized that: (1) the biodistribution of LNPs to the liver and other organs is likely to be different for uncharacterized reasons, (2) an acid dissociation constant (pKa) close to 6.4 of amine of an ionizable cationic lipid is required for the efficient translocation of LNPs from the endosome to cytosol, and (3) absorption of LNPs by the liver is, at least in part, mediated by apolipoprotein E-mediated modification in the blood [67]. Due to limited data, a series of different lipids has been synthesized, and their tissue targeting has been examined.

### 7.1. Dendrimers

A dendrimer is a globular synthetic chemical with branched hydrophobic moiety. According to the SORT hypothesis, a series of novel ester-based dendrimers, such as 5A2-SC8, have been synthesized [67,68] (Table 6). During the course of a series of these studies, the authors noticed that an increase in DOTAP enhanced the targeting of LNPs to the lung [31]. Separately, it was also found that an inclusion of C18 phosphatidic acid enhanced LNP targeting to the spleen [67,68]. Currently, these are known as SORT molecules, which modulate the targeting property of LNPs. More recently, researchers have explored the possibility of a novel class of alkenyl-thiols [31] and multi-tailed ionizable phospholipids [69] for expanding the SORT hypothesis. For organ cell specificity, a liver-directed formulation mainly delivered LNP-mediated genes into hepatocytes. In contrast, 66% of pulmonary endothelial cells expressed exogenous genes. In the spleen, LNP-mediated genes were expressed in 20% of macrophages, 12% of B cells, and 10% of T cells [68].

In a separate formulation of LNPs by Dahlman and colleagues, more than 50% of splenic endothelial cells were able to express exogenous genes [70]. In this experiment, each LNP was individually barcoded, and a mixture encapsulating Cre mRNA was administered to Ai14 mice. Then, fluorescence-active cells in the spleen were FACS-sorted based on the expression of CD31, an endothelial marker. The researchers found an accumulation of a lipid 7C3 in these cells, concluding that this formulation provided the selective LNP capable of consistently delivering exogenous genes to the spleen. To estimate the efficiency of gene delivery by this LNP, these authors chose ICAM-2, an abundantly expressed surface protein on endothelial cells, and examined the efficiency of genome editing by CRISPR/Cas9. They found that 13–20% of indel were observed in these splenic endothelial cells, as well as hepatocytes, indicative of a shared mechanism of cellular incorporation between them.

### 7.2. Polymers

Several studies have tested the possibility of polyesters for extrahepatic targeting of LNPs [71]. Based on the screening of a library with 1200 candidates, the best formulation (PE4k-A17-0.2C8) with an optimal length of polymer, amine, and LNP composition based on the intensity of luciferase was uncovered [72]. Overall, the selected nanoparticle has a neutral surface charge, a diameter of approximately 100 nm, a pKa between 7.5 and 8.0, and more than 90% of RNA-binding activity. This compound exclusively delivered Cre mRNA to the lung, as demonstrated by tdTomato fluorescence mice. Besides its detailed mechanism of pulmonary targeting, this excellent targeting specificity for the lung provides a further starting point for the structure-activity study of LNPs.

### 7.3. Antibody-Modified LNPs

A study has examined the treatment of cardiac fibrosis by targeting LNPs to T cells [73]. In this study, LNP-encapsulated mRNA for a chimeric antigen receptor (CAR) against the fibrosis activation protein (FAP) was first delivered to T cells via anti-CD5 antibody-modified LNP. The expressed FAP-CAR in the T cells then reacts with FAP in a diseased fibroblast, leading to a therapeutic outcome.

## 8. Future Perspective

Current LNP technology has an advantage in the encapsulation of larger mRNA for therapeutic use compared to AAV. Furthermore, LNPs are capable of delivering a gene into non-dividing cells. Specifically, for liver disease, there are multiple examples where LNP-based therapy has proven effective in preclinical and clinical studies (Table 4 and Table 5). However, there are also limitations of LNP at this stage discussed below. For example, biodegradation of LNP is one such issue to be addressed. Specifically, one current problem with LNPs is that several lipids are less susceptible to being biologically degradable; thus, these lipids create a concern of hepatotoxicity. In an attempt to accelerate their effective biodegradation, regardless of molecular weight and chemical nature, the introduction of ester and thioester into these compounds may prove beneficial.

## Figures and Tables

**Table 1:** Lipid composition of FDA-approved LNP.

Product	Lipid Composition	Ref
	**Cationic ionizable lipid/helper phospholipid/cholesterol/PEG-lipid**	
Onpattro	DLin-MC3-DMA/DSPC/Chol/DMG-PEG2000 = 50/10/38.5/1.5	[5]
mRNA-1273	SM-102/DSPC/Chol/DMG-PEG2000 = 50/10/38.5/1.5	[6]
BNT126b2	ALC-0315/DSPC/Chol/ALC-0159 = 46.3/9.4/42.7/1.6	[7]

**Table 2:** Comparison of preparation and modification procedure for mRNA and sgRNA.

	mRNA	sgRNA
Preparation	In vitro transcription	Solid phase synthesis
Modification		
5′-Capping	Enzymatic	Not applicable
2′-O-methylation	Enzymatic	Synthetic
3′/5′-O-phosphorothioation	Not applicable	Synthetic
Replacement by uridine analog	Enzymatic	Not applicable
Codon optimization	In silico	Not applicable

**Table 3:** 5′-Capping of mRNA [14].

Nomenclature	5′-5′ Bridge	Base				Enzyme
		Non-Transcribed Nucleoside	1st Transcribed Nucleoside		2nd Transcribed Nucleoside	
		Guanine	Ribose 2′-	Adenine	Ribose 2′-	
Uncapped	GpppA/G	No modification	OH	H	OH	
Cap 0	GpppA/G	N7-Me	OH	H	OH	TGS
Cap 1	GpppA/G	N7-Me	Me	H	OH	CMTR1
Cap 2	GpppA/G	N7-Me	Me	H	Me	CMTR2
	GpppG	N7-Me; N2-Me; N2,2′-di-Me	OH	H	OH	TGS
	GpppA	N7-Me	OH	N2-Me	OH	SAMT
	GpppA	N7-Me	Me	N6-Me	Me	CAPAM

TGS, 2,2,7-trimethylguanosine synthase; CAMT, cap-specific mRNA (nucleoside-2′-O-)-methyltransferase; CAPAM, cap-specific adenosine N6-methyltransferase; SAMT, S-adenosylmethionine transferase.

**Table 4:** LNP-based preclinical studies.

Disorders	Target	Genetic Material	Ref
Bleeding disorder	FVII	siRNA	[41]
Hypercholesterolemia	Pcsk9	siRNA	[23]
Hypercholesterolemia	ApoB	siRNA	[40]
Transthyretin-mediated amyloidosis	Ttr	siRNA	[24]
Transthyretin-mediated amyloidosis	Ttr	siRNA	[23]
Anemia	mEPO, pEPO	Therapeutic mRNA	[26]
Anemia	hEPO	Therapeutic mRNA	[27]
Anemia	hEPO	Therapeutic mRNA	[28]
Fabry disease	hGLA	Therapeutic mRNA	[42]
Fabry disease	hGLA	Therapeutic mRNA	[43]
HIV	anti-HIV-1 antibody	Therapeutic mRNA	[44]
DMD	Human DMD exon 45	Cas9	[45]
Glioblastoma	Plk1	Cas9	[46]
Hemophilia A and B	Serpinc1 (encoding antithrombin)	Cas9	[47]
HIV	TatDE	Cas9	[48]
Hypercholesterolemia	Angptl3	Cas9	[49]
Hypercholesterolemia	Pcsk9	Cas9	[24]
Hypercholesterolemia	Pcsk9	Cas9	[31]
Hypercholesterolemia	Pcsk9	Cas9	[32]
Transthyretin-mediated amyloidosis	Ttr	Cas9	[33]
Hypercholesterolemia	Pcsk9	ABE	[36]
Hypercholesterolemia	Pcsk9	ABE	[35]
Phenylketonuria	Pah	CBE	[37]
Tyrosinemia I	Fah	ABE	[38]

**Table 5:** Clinical trials of LNP-mediated gene therapy.

NCT Number	Conditions-1	Conditions-2	Target	Payload	Phases	Status	Sponsor/Collaborators	Ref
NCT04601051	Amyloidosis	hATTR	Transthyretin	Cas9 mRNA	Phase 1	Recruiting	Intellia Therapeutics	[61]
NCT01960348	Amyloidosis	hATTR	Transthyretin	siRNA	Phase 3	Completed	Alnylam Pharmaceuticals	[5,56,62,63,64,65]
NCT01437007	Cancer	Hepatic Metastases	PLK1	siRNA	Phase 1	Completed	NCI; NIH Clinical Center	
NCT03323398	Cancer	Solid Tumor	Human OX40L	mRNA	Phase 1/2	Terminated	ModernaTX, Inc.	
NCT03739931	Cancer	Solid Tumor	Human OX40L, IL-23, and IL-36γ	mRNA	Phase 1	Recruiting	ModernaTX, Inc.; AstraZeneca	[66]
NCT04675996	Cancer	Solid Tumor	miR-193a-3p	miRNA	Phase 1	Recruiting	InteRNA	
NCT02110563	Cancer	Solid Tumor	MYC	siRNA	Phase 1	Terminated	Dicerna Pharmaceuticals, Inc.	
NCT02314052	Cancer	Hepatocellular Carcinoma	MYC	siRNA	Phase 1/2	Terminated	Dicerna Pharmaceuticals, Inc.	
NCT04486833	Cancer	Carcinoma, Non-Small Cell Lung	TUSC2	DNA plasmid	Phase 1/2	Recruiting	Genprex, Inc.	
NCT05062980	Cancer	Non-Small Cell Lung Cancer	TUSC2	DNA plasmid	Phase 1/2	Recruiting	Genprex, Inc.	
NCT05497453	Cancer	Solid Tumor	2 independent epigenomic controllers	biscistronic mRNA	Phase 1/2	Recruiting	Omega Therapeutics	
NCT02227459	Hepatic Fibrosis		HSP47	siRNA	Phase 1	Completed	Bristol-Myers Squibb; Nitto Denko Corporation	
NCT04416126	OTCD		Ornithine transcarbamylase	Therapeutic mRNA	Phase 1	Completed	Arcturus Therapeutics, Inc.	
NCT04442347	OTCD		Ornithine transcarbamylase	Therapeutic mRNA	Phase 1	Recruiting	Arcturus Therapeutics, Inc.	
NCT05526066	OTCD		Ornithine transcarbamylase	Therapeutic mRNA	Phase 2	Recruiting	Arcturus Therapeutics, Inc.	

OTCD, Ornithine transcarbamylase deficiency; hATTR, hereditary transthyretin amyloidosis.

**Table 6:** Tissue targeting of LNP.

Target Organ	Basic Component	Additive	Ref
	**Cationic ionizable lipid/helper phospholipid/cholesterol/PEG-lipid**	**(SORT molecule)**	
Conventional	LP01/DSPC/Chol/PEG2000-DMG = 45/9/44/2	Not added	[33]
	DLin-MC3-DMA/DSPC/Chol/DMG-PEG2000 = 50/10/38.5/1.5	Not added	[69]
	5A2-SC8/DOPE/Chol/C14PEG2000 = 23.8/23.8/47.8/4.8	Not added	[67]
Liver	5A2-SC8/DOPE/Chol/C14PEG2000 = 19/19/38.4/4	DODAP = 20	[67,68]
	9A1P9/5A2-SC8/Chol/DMG-PEG2000 = 25/30/30/1	Not added	[69]
Spleen	5A2-SC8/DOPE/Chol/C14PEG2000 = 16.7/16.7/33.3/3.3	18:1 PA = 30	[67,68]
Lung	5A2-SC8/DOPE/Chol/C14PEG2000 = 11.9/11.9/23.8/2.4	DOTAP = 50	[67,68]
	9A1P9/DDAB/Chol/DMG-PEG2000 = 60/30/40/0.4	Not added	[69]
	5A2-SC8/DOPE/Chol/DMG-PEG = 15/15/30/3	DOTAP = 63	[31]

18:1 PA, 1,2-dioleoyl-sn-glycero-3-phosphate; DODAP, 1,2-dioleoyl-3-dimethylammonium propane; DOTAP, 1,2-dioleoyl-3-trimethylammonium-propane; SORT, selective organ targeting.

## Data Availability

Not applicable.
